# Case report: Intradural-extramedullary cervical spine clear cell meningioma mimicking a schwannoma in a child

**DOI:** 10.3389/fonc.2024.1505141

**Published:** 2025-01-10

**Authors:** Xiang Yang, Chongxi Xu, Seidu A. Richard, Yongliang Jiang, Jiaxi Wang, Bin Xu, Jianguo Xu, Hao Li

**Affiliations:** ^1^ Department of Neurosurgery, West China Hospital, Sichuan University, Chengdu, Sichuan, China; ^2^ Department of Biochemistry and Forensic Sciences, School of Chemical and Biochemical Sciences, C. K. Tedam University of Technology and Applied Sciences (CKT-UTAS), Navrongo, Ghana

**Keywords:** case report, meningioma, pain, spine, radiotherapy

## Abstract

Clear cell meningioma (CCM) is an exceedingly rare subtype of meningioma, with spinal occurrences being even more uncommon. It predominantly affects children and is characterized by a high recurrence rate and poor prognosis, posing significant challenges for clinical treatment. Currently, gross total resection (GTR) is the best approach to reduce recurrence and improve prognosis in these patients. However, detailed descriptions of intraoperative findings, particularly for intraspinal cervical CCM, are scarce in the literature. Here, we report a rare case in which the upper cervical spinal CCM mimicked a schwannoma, detailing the surgical treatment strategy and prognosis. Additionally, we analyzed all previously reported cases of spinal CCM to investigate the clinical characteristics, optimal treatment strategies, and prognostic factors, which may be of particular interest to neurosurgeons.

## Introduction

According to existing literature, the incidence of meningiomas worldwide is approximately 3-4 cases per 100,000 people annually, and meningiomas account for about 15-20% of all intracranial tumors. Among them, CCM represents less than 1% of all meningioma cases., they predominantly affects children. Pathologically, it is characterized by clear polygonal cells that are rich in glycogen (1, 2). Since the first case of CCM was reported in 1990, an increasing number of cases have shown that, compared to ordinary meningiomas, this type of tumor has a higher local recurrence rate and a more aggressive clinical course (3, 4). For these reasons, the World Health Organization (WHO) classified CCM as a Grade II tumor in 2016 (5).Intraspinal CCM are rarer than their intracranial counterparts (6). Moreover, in younger patients, these tumors may exhibit more aggressive behavior and are associated with a poorer prognosis (7), and tend to have a higher recurrence rate (8). Unlike intracranial CCM, the role of adjuvant radiotherapy following surgery for intraspinal CCM remains controversial. Current viewpoint suggests that for patients with intraspinal CCM, immediate postoperative radiotherapy may not be necessary following gross total resection, particularly in pediatric patients, owing to their lower tolerance for the side effects of radiotherapy (9–11). To date, GTR of intraspinal CCM is considered the optimal treatment for reducing recurrence rates and achieving longer progression-free survival (PFS) and overall survival (OS) (10, 12). Currently, the literature reports that intraspinal CCM predominantly occurs in the lumbar spine (6) with very fewer cases involving the upper cervical spine. Additionally, the role of radiotherapy in the management of intraspinal CCM remains controversial, particularly in pediatric patients, there still lack of detailed case reports on the treatment outcomes of this rare condition, especially in cases without dural attachment. Here, we present a case of a ventral upper cervical CCM without dural attachment, providing a detailed account of its clinical features, imaging characteristics, and intraoperative details, along with long-term follow-up results. Also, we conduct a literature review of reported cases of spinal canal CCM to explore diagnosis, treatment, and prognosis.

## Case report

### Clinical history and physical examination

A 6-year-old male presented with a one-month history of intermittent neck pain, which progressively restricted movement in all directions. General physical examination and neck assessment revealed no significant abnormalities. However, there was marked tenderness on palpation of the cervical spine from C1 to C4. Sensory examination and reflexes in the upper and lower extremities, as well as the abdomen, were normal. Muscle strength was 5/5, and muscle tone was normal in both the upper and lower extremities.

### Imaging findings

**Figure 1:**
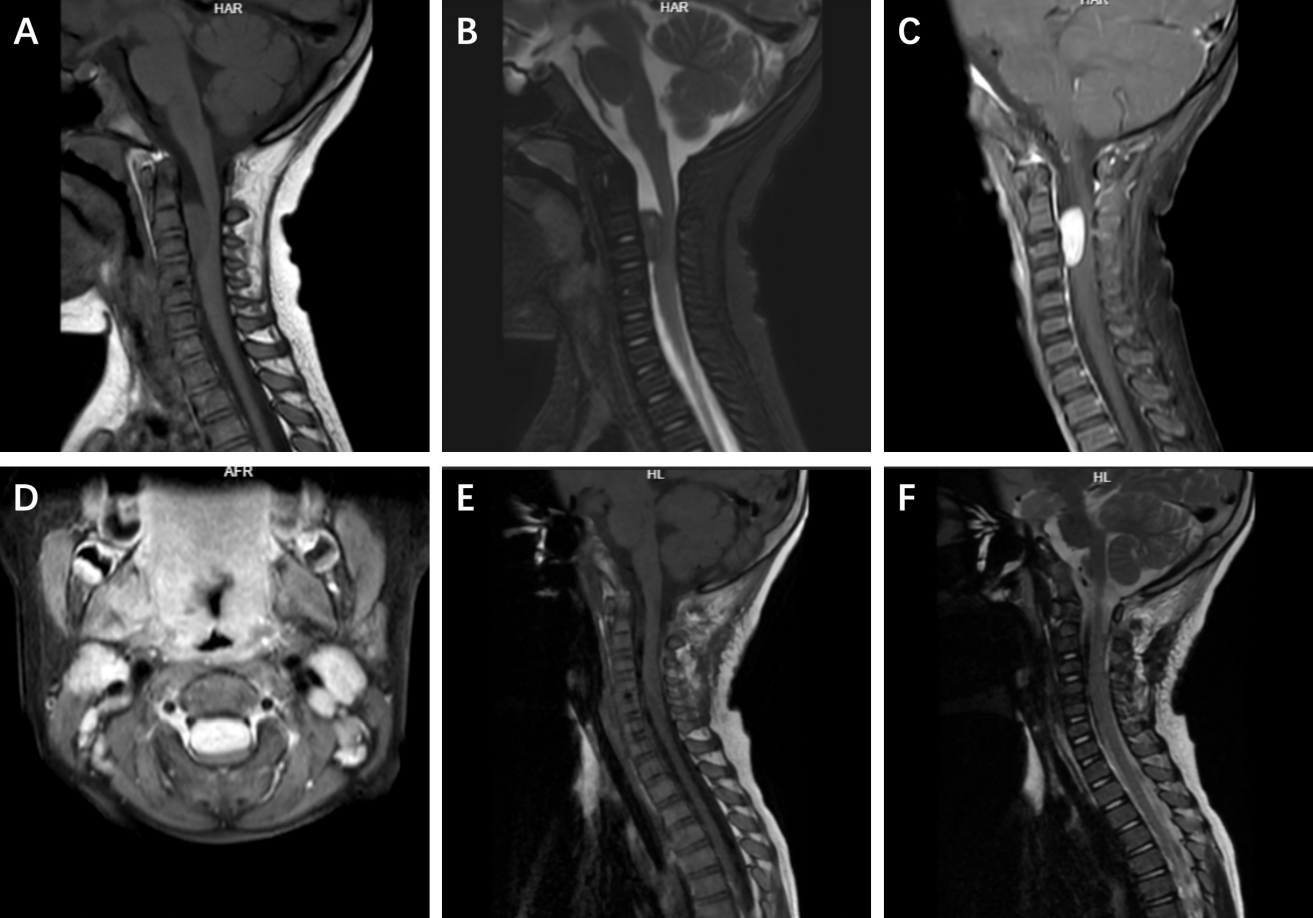
The preoperative and postoperative imaging findings of the patient. On preoperative MRI, the lesion showed variable signal intensity within the spinal canal on sagittal T1-weighted and T2-weighted images **(A, B)**. Uniform enhancement was observed on sagittal and axial views **(C, D)**. Postoperative sagittal T1-weighted and T2-weighted images showed signal intensity consistent with no tumor recurrence **(E, F)**.

The results of routine laboratory investigations were within normal ranges. Cervical spine magnetic resonance imaging (MRI) revealed a space-occupying lesion measuring approximately 4 × 3 × 2 cm within the C3–4 spinal canal, without a “dural tail sign”, displacing the spinal cord posteriorly. On T1- and T2-weighted images (WIs), the mass appeared iso-dense and showed homogeneous enhancement with gadolinium contrast (**Figures 1A–D**). On the basis of the radiological findings and the clinical findings, a preliminary diagnosis of schwannoma was made, with differential diagnoses including meningioma. With no contraindications for surgery, the patient was scheduled for surgery the following day.

### Operation

**Figure 2:**
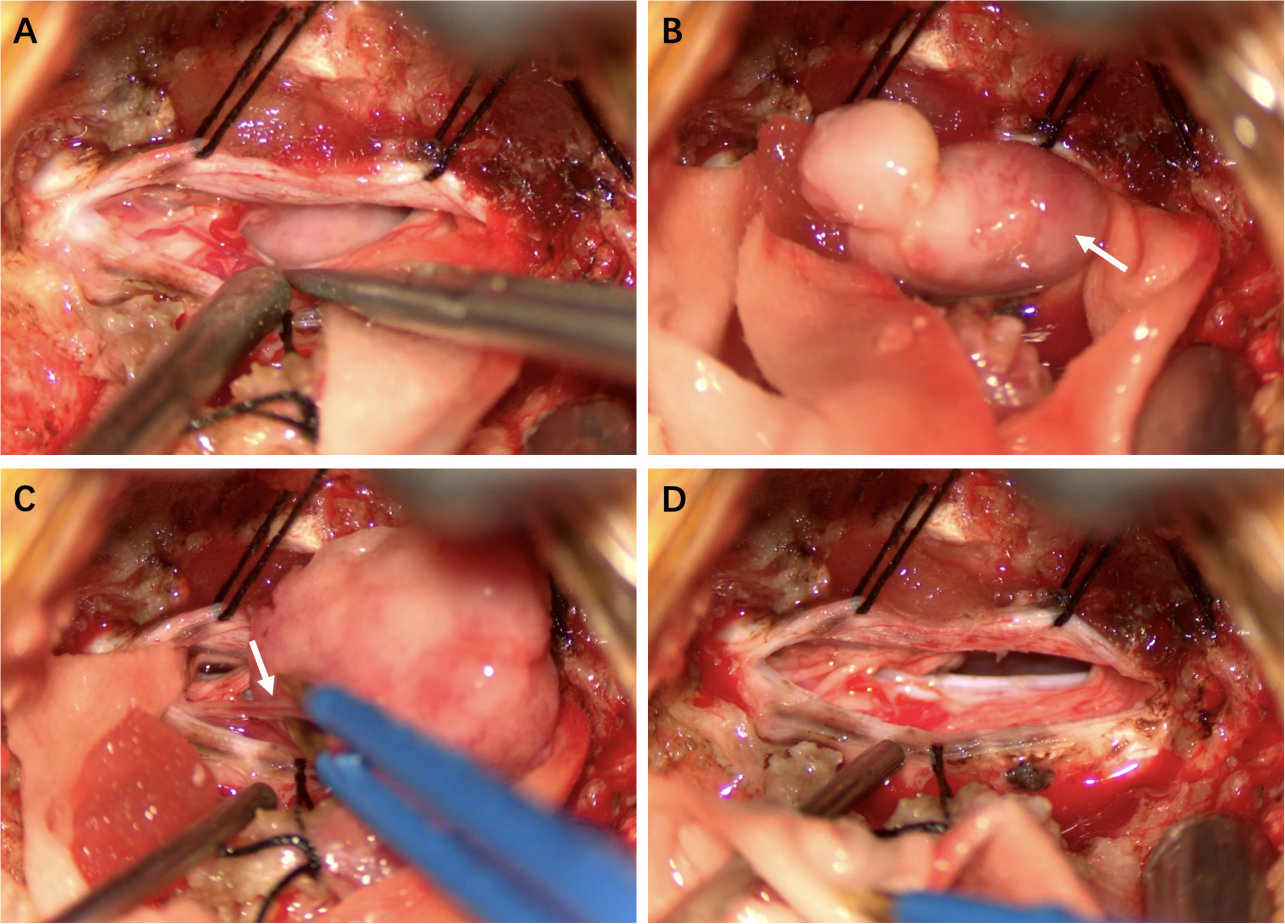
Intraoperative Findings **(A)** Tumor exposure. **(B)** The tumor capsule was intact and mobile, with no significant attachment to the dura mater. **(C)** Some nerves were fused with the lesion. **(D)** Complete tumor resection.

To avoid bilateral spinal cord damage, a midline posterior cervical incision was made to expose the right lamina of C2 and C3. The intradural-extramedullary lesion was located in the C2–3 spinal canal and was positioned ventrally, causing severe posterior displacement and compression of the spinal cord. After the lesion from the right side of the spinal cord was exposed, it was unexpectedly found that the lesion was mobile and had no significant attachment to the dura mater. The mass was tough, with a yellowish-white appearance. It adheres tightly to multiple nerve roots, with some nerves partially fused to the lesion. We first finished the intratumoral decompression, meticulously separated the tumor from the nerve roots, and then cut off the so-called nerve of lesion origin under electrophysiological monitoring to achieve complete resection of the lesion (**Figure 2**).

### Pathological results

**Figure 3:**
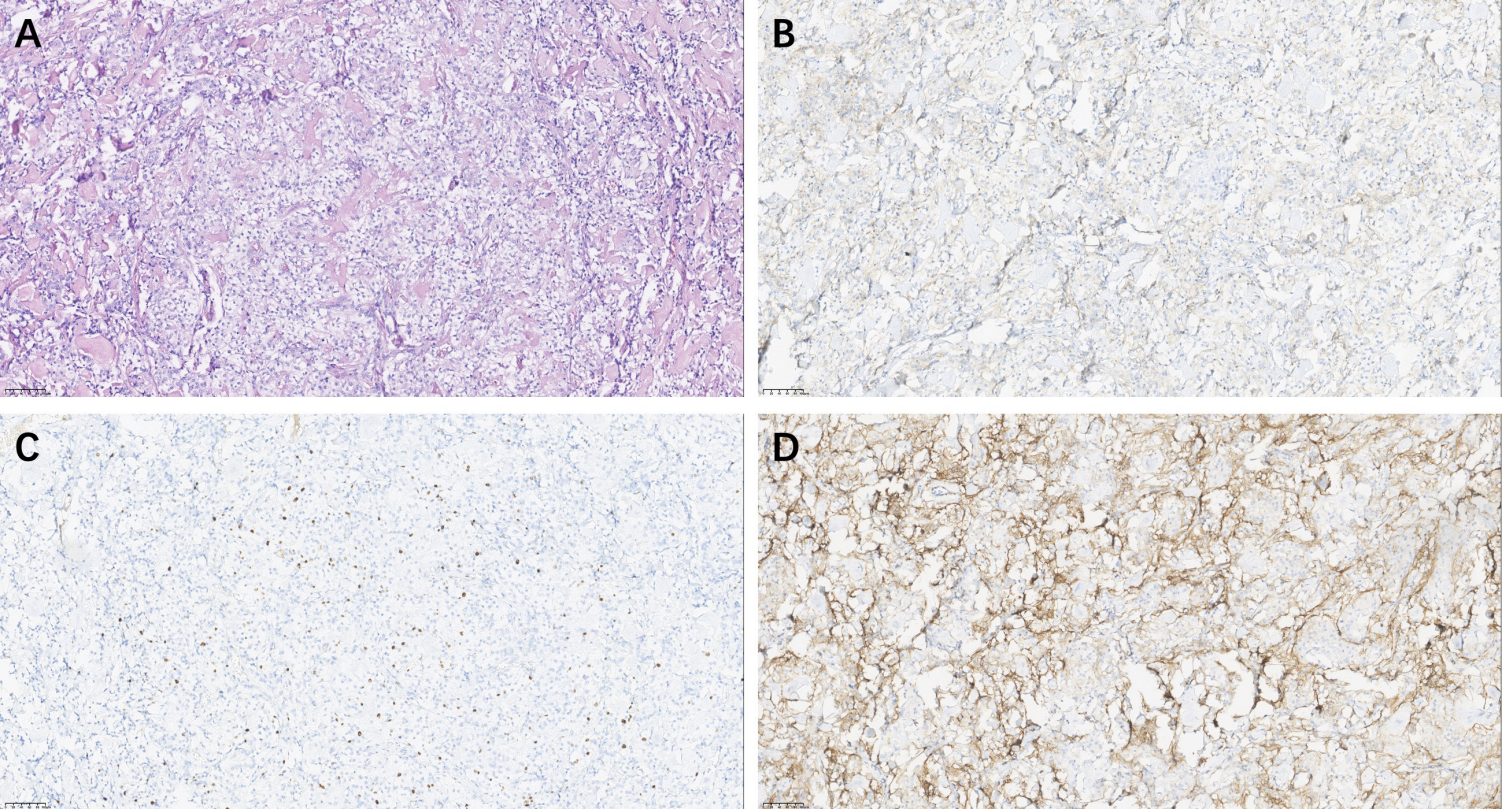
Pathological Analysis of CCM. Hematoxylin & eosin (magnification, × 20) **(A)**; Epithelial Membrane Antigen positive: EMA (magnification, × 20) × **(B)**, Ki-67 (MIB-1) Immunostaining (magnification, × 20) **(C)**, Somatostatin Receptor 2 staining (magnification, × 20) **(D)**.

Histopathological analysis revealed polygonal cells with clear cytoplasm rich in glycogen. Immunohistochemistry (**Figure 3**) of the tumor revealed positivity for epithelial membrane antigen (EMA) and somatostatin receptor-2 (SSTR-2), with a Ki-67 (MIB-1) labeling index of 5%. These findings supported the diagnosis of CCM.

### Postoperative course

The postoperative course was uneventful, and postoperative MRI confirmed total resection of the lesion (**Figures 1E, F**). The child was discharged one week after surgery. Given that young children cannot tolerate radiotherapy, postoperative radiotherapy was not given to the child. There were no signs of recurrence during the two years of follow-up.

## Discussion

CCM has a higher recurrence rate and more aggressive biological behavior than typical meningioma (13). And has potential for metastasis and associated low progression-free survival rates (14). Unlike typical meningioma, which commonly occur in the thoracic spine, spinal CCM tends to involve the lumbar spine, with some cases showing evidence of bone destruction (15). Clinically, spinal CCM presents similarly to other intradural extramedullary tumors and lacks specific distinguishing features. Symptoms are typically related to the tumor’s location, size, and extent of its involvement with the spinal cord or nerve. Genetic studies have shown that besides NF2 mutations, loss of SMARCE1 may also associated with CCM. However, additional studies are needed to clarify the mechanisms underlying SMARCE1-related clear cell meningioma development (16). MRI is an effective preoperative diagnostic tool for spinal CCM. According to the literature, the imaging characteristics of most intradural CCMs resemble those of typical meningioma (14). They typically present as well-demarcated, round or oval intradural-extramedullary masses with isointense signals on both T1- and T2-weighted images. These tumors commonly display a broad dural attachment and show homogeneous enhancement after gadolinium administration, with some patients exhibiting a dural tail sign. However, some cases lack of dural attachment (17), making preoperative MRI prone to misdiagnosis as “schwannoma”. In addition, these tumors may exhibit aggressive features such as vertebral body destruction or invasion of the intervertebral foramen, mimicking malignant tumors (15, 18). Additionally, there have been reports of intramedullary CCMs (19). In summary, preoperative imaging lacks specificity, and definitive diagnosis still relies on postoperative pathological examination.

Current research suggests that the postoperative recurrence rate of intraspinal CCM can reach 40% (20). Therefore, reducing the recurrence rate and prolonging PFS are significant challenges for neurosurgeons. Chaim et al. suggested that adjuvant radiotherapy may help lower the recurrence risk in patients with a high Ki-67 index (21). Kobayashi et al. proposed that en bloc resection of the tumor without disrupting the capsule during surgery can prevent the dissemination of tumor cells. Furthermore, they identified positive PR expression via postoperative pathology as a potential predictor of favorable prognosis (22). Compared to conventional CCM, non-dural attached intradural spinal CCMs are more easily completely resected. Zhang et al. suggest that the recurrence rate of this specific type of meningioma is lower than that of other types. Nevertheless, adjuvant radiation therapy is still recommended (6). However, there is still no consensus on whether routine postoperative radiotherapy should be applied after GTR in pediatric patients (11).

We reviewed all well-documented cases of intraspinal CCM (**Supplementary Table S1**), and analyzed factors such as patient age, sex, imaging characteristics, extent of surgical resection, postoperative pathology, and recurrence. We found that intraspinal CCMs typically involve the lumbar spine, particularly the cauda equina, while cervical spine involvement is rare. To date, fewer than five cases involving the cervical spine have been reported. Here, we report a case of an intradural-extramedullary cervical spine CCM in a child, which were the second well-documented case of cervical CCM in a pediatric patient, and the first case where the lesion was located on the ventral side of the upper cervical spine, mimicking a schwannoma both on preoperative imaging and under the surgical microscope. The World Federation of Neurosurgical Societies (WFNS) does not have specific guidelines exclusively for adjuvant radiotherapy in CCM. However, general recommendations for meningiomas of WHO grade II, which includes CCM, suggest considering adjuvant radiotherapy when GTR is not achieved. This approach is recommended to reduce recurrence rates. In CCM, the decision to use adjuvant radiation typically depends on tumor resectability and recurrence risk, especially when surgical margins are compromised or if there is nodular recurrence. In this case, given the patient’s pediatric age and complete encapsulated resection, adjuvant radiotherapy was not administered. The patient was followed for two years with no evidence of tumor recurrence.

This case report provides valuable insights into the rare occurrence of intradural-extramedullary cervical spinal CCM in a pediatric patient, contributing to the limited literature on such patients. However, the case is limited by its rarity, limiting generalizability, and the absence of preoperative genetic profiling, which could provide further insight into the molecular mechanisms of CCM. Additionally, while the two-year follow-up period is promising, a longer follow-up would be essential to fully evaluate long-term recurrence risk. More detailed radiological findings and further research on genetic markers are needed to enhance understanding and management of CCMs.

## Patient perspective

I was diagnosed with a rare condition, intraspinal CCM, located in my cervical spine. Initially, the doctors were unsure of the diagnosis, as the tumor resembled a schwannoma on the MRI images. It was a very overwhelming experience for me and my family, as we were concerned about what this might mean for my future health. However, after undergoing surgery, the tumor was completely removed, and the doctors were confident about the success of the procedure. The surgery was challenging, but I felt well-supported by the medical team throughout the process. They explained everything clearly, making sure I understood the procedure and recovery steps. Post-surgery, I had some discomfort and fatigue, but it was manageable. Fortunately, because my tumor was fully encapsulated and I was a child, the decision was made not to undergo radiotherapy. This relieved me of some concerns about the side effects of radiation, especially since I was already recovering from surgery. Over the next two years, I had regular check-ups, and I am happy to report that I have not experienced any tumor recurrence. I feel incredibly fortunate that I was able to receive the best care possible. Looking back, it has been a challenging but ultimately positive journey, and I am hopeful for my future. For other patients facing similar situations, I would encourage them to stay hopeful, trust their medical team, and stay focused on recovery.

## Conclusion

Intraspinal CCM, particularly in the pediatric upper cervical spine, is extremely rare, with only a few cases reported in the literature. However, it should be considered an important differential diagnosis for spinal intradural lesions. This is because both preoperative imaging and intraoperative microscopic examination can lead to misdiagnosis of schwannoma. Despite its aggressive nature and high recurrence rate, the role of adjuvant radiotherapy post-surgery remains controversial. Currently, complete surgical resection and vigilant follow-up are essential for managing these patients.

## Data Availability

The original contributions presented in the study are included in the article/**Supplementary Material**. Further inquiries can be directed to the corresponding author.
