# Supplementary material for: Case report: Intradural-extramedullary cervical spine clear cell meningioma mimicking a schwannoma in a child
Source: Front Oncol. 2025 Jan 10;14:1505141. doi: 10.3389/fonc.2024.1505141 (PMC11758182; doi:10.3389/fonc.2024.1505141)
Supplement: Supplementary file 1 [file Table1.docx]

Supplemental table 1: Summary of all well-documented intraspinal CCM.

| **Case** | **Age/sex** | **Location** | **EOR** | **Adjuvant**  **treatment** | **Encapsulation**  **and Root involvement** | **Ki-67** | **Time to recurrence/**  **location** | **Follow-up time** |
| --- | --- | --- | --- | --- | --- | --- | --- | --- |
| Zorludemir,1995^1^ | 17y, F | L4-5 | GTR | No | Encapsulation (+), Root (+) | Unreported | No | 36 m |
|  | 23y, F | L5 | GTR | No | Unreported | Unreported | No | Unreported |
|  | 38y, M | Sacrococcygeal | GTR | No | Unreported | Unreported | Four recurrences | Unreported |
|  | 34y, M | L4-S1 | GTR | No | Unreported | Unreported | 12m./ Unreported | Unreported |
|  | 36y, F | L2-5 | GTR | No | Unreported | Unreported | No | Unreported |
| Holtzman,1996^2^ | 32y, M | L3-4 | GTR | No | Encapsulation (+), Root (+) | Unreported | No | 1 m |
| Pimentel, 1996^3^ | 55y, M | Cervical | GTR | No | Unreported | Unreported | No | Unreported |
|  | 21y, F | Lumbar | GTR | No | Unreported | Unreported | No | Unreported |
| Prinz,1996^4^ | 38y, M | Sacrococcygeal | GTR | No | Unreported | Unreported | Unreported/ Multiple recurrences | Unreported |
| Matsui,1998^5^ | 9y, F | T11–12, L2 and  L4–5 | GTR | No | Encapsulation (+), Unreported | 12% | No | 1 y |
| Dubois.1998^6^ | 10y, F | L1–4， cauda equina | GTR | No | Encapsulation (+), Unreported | Unreported | 6m/ L1, L2 | 1 y |
| Maxwell,1998^7^ | 31y, F | L3 | GTR | No | Unreported, Root (+) | Unreported | Unreported | Unreported |
| Cances,1998^8^ | 9 y, F | Lumbar | GTR | Yes | Unreported | Unreported | Recurrence at 5 months/same location | 5 m |
| Heth, 2000^9^ | 7 y, F | L4–5 | GTR | No | Unreported | Unreported | No | 13 m |
| Jallo, 2001^10^ | 22m, F | C3–4 | STR | Yes | Unreported | Unreported | 3m/same location; 2 y/Metastatis  to post fossa | 3 y |
| Carrà,2001^11^ | 22m, M | T11- L4 | GTR | No | Encapsulation (+), Unreported | Unreported | No | 4 y |
| Florman, 2001^12^ | 20y, m | L4–5 and L5-S1.  Multifocal. | GTR | No | Encapsulation (+), Root (+) | Unreported | Unreported | Unreported |
| Yu,2002^13^ | 14m, F | T12-L2 | GTR | Yes | Encapsulation (+), Unreported | 3 % | 8m/T12; 15m/T12 | 23 m |
| Boet, 2003^14^ | 34, M | L4-S3 | STR | Yes | No, Root (+) | Unreported | No | 15 m |
| Chen,2004^15^ | 41y, F | L4-5 | GTR | No | Encapsulation (+), Root (+) | Unreported | No | 4 m |
| Payano,2004^16^ | 24y, M | L3-4 | GTR | No | Encapsulation (+), Root (+) | ＜1% | No | 52 m |
|  | 19y, F | L3 | GTR | No | Encapsulation (+), Root (+) | ＜1% | No | 61m |
| Oviedo,2005^17^ | 7y, M | L2-3 | GTR | No | Encapsulation (+), Root (+) | 10% | No | 1 y |
| Epstein,2005^18^ | 41y, F | L3-L4 | GTR | No | Encapsulation (+), Root (+) | 2–3% | No | 6 m |
| Dhall, 2005^19^ | 32y, F | T12-S1 | GTR | No | Encapsulation (-), Unreported | Unreported | No | 4 y |
| Liu, 2005^20^ | 2.2y, M | T10-L1 | GTR | No | Encapsulation (+), Unreported | Unreported | No | 60 m |
| Jia,2005^21^ | 40y, F | L1–2 | GTR | No | Encapsulation (+), Root (+) | Unreported | No | 6 m |
| Park, 2006^22^ | 65y, F | T9-10 | GTR | Yes | Unreported | Unreported | No | 2 y |
| Vural,2007^23^ | 4y, F | C1-2 | GTR | No | Unreported | Unreported | No | 6 m |
| Salehpour,2008^24^ | 21y, M | Cervicomedullary  junction to C3 | GTR | No | Unreported | Unreported | No | 3 y |
| Nakajima,2009^25^ | 21y, F | L2-3 | GTR | No | Encapsulation (+), Root (+) | Unreported | No | 6 m |
| Colen, 2009^26^ | 13y, F | L4-L5 | GTR | Yes | Unreported | 5–7% | No | 2 y |
| Wu,2009^27^ | 35y, F | C7 | GTR | No | Unreported | 10-40% | No | short  period |
| Ko,2011^28^ | 34y, F | T12-L2 | GTR | No | Encapsulation (+), Root (+) | Unreported | No | 2 Y |
| Kobayashi,2012^29^ | 43y, M | L1–3 | GTR | No | Encapsulation (+), Root (+) | 5 % | No | 7 y |
| Zhang,2012^30^ | 26y, F | T12-L1 | GTR | No | Encapsulation (+), Unreported | Unreported | No | 1 M |
| Balogun,2013^31^ | 3 y, M | L2-5 | GTE | Yes | Encapsulation (-), Root (+) | 5–10% | 9m/ L1–L2 | 9m |
| Wang,2014^32^ | 79y, M | C1-2 | GTR | No | Unreported | Unreported | Unreported | Lost to follow-up |
|  | 18y, M | C6-T2 | GTR | Yes | Unreported | Unreported | 19m/same location; 12m/ same location; 9m/C7-T2 | 49 m |
|  | 35y, M | L5-S1 | GTR | Yes | Unreported | Unreported | No | 1 m |
| Li, 2016^33^ | 21 y, M | L5 | GTR | No | Unreported | 5–15 % | No | 5 m |
|  | 43y, F | L3-S3 | STR | No | Unreported | 5 % | No | 10 m |
|  | 7y, F | T11-L1 | GTR | No | Unreported | 20 % | No | 9 m |
|  | 7y, F | L2-4 | GTR | No | Unreported | 10 % | No | 2 y |
|  | 4Y, M | T11-12 | GTR | No | Unreported | Unreported | No | 2 y |
|  | 20y, F | L4-5 | GTR | No | Unreported | 5–10 % | No | 2 m |
| Kawasaki, 2018^34^ | 8y, F | L3 | GTR | No | Encapsulation (+), Root (+) | 26% | No | 24 m |
| Inoue,2018^35^ | 5y, M | L5-S1 | GTR | No | Encapsulation (+), Root (+) | 3% | No | 7m |
| Li, 2019^36^ | 15y, F | L3 | GTR | No | Unreported | Unreported | No | Unreported |
|  | 16y, M | L5–S1 | GTR | No | Unreported | Unreported | No | Unreported |
|  | 16y, F | T11–L1 | STR | Yes | Unreported | Unreported | Yes/ Unreported | Unreported |
|  | 14y, F | L4 | STR | Yes | Unreported | Unreported | Yes/ Unreported | Unreported |
| Zhang, 2021^37^ | 45, F | L3 | GTR | Yes | Encapsulation (+), Root (+) | 10% | No | 12 m |
| Alsadiq,2021^38^ | 25y, F | L4-5 | STR | No | Encapsulation (+), Root (+) | 2 % | Unreported | Unreported |
| Maamri,2022^39^ | 58y, F | L3 | GTR | No | Encapsulation (+), Root (+) | Unreported | No | Unreported |

**GTR: gross total resection; STR: Subtotal Resection; RT: Radiotherapy; Encapsulation:** **Existence of a capsule as confirmed in the literature; y: years; m: month.**

**Reference**

1. Zorludemir S, Scheithauer BW, Hirose T. Clear cell meningioma: a

clinicopathologic study of a potentially aggressive variant of

meningioma. Am J Surg Pathol 1995;19:493 - 505..

2. Holtzman, R. N. N. & Jormark, S. C. Nondural-based lumbar clear cell meningioma: Case report. *J. Neurosurg.* **84**, 264–266 (1996).

3. Pimentel J, Fernandes A, Pinto AE, Fonseca I, Moura Nunes JF, Lobo

Antunes J. Clear cell meningioma variant and clinical aggressiveness.

Clin Neuropathol 1998;17:141- 6..

4. Prinz M, Patt S, Mitrovics T, Cervos-Navarro J. Clear cell meningioma:

report of a spinal case. Gen Diagn Pathol 1996;141:261 - 7.

5. Matsui, H., Kanamori, M., Abe, Y., Sakai, T. & Wakaki, K. Multifocal clear cell meningioma in the spine: A case report. *Neurosurg. Rev.* **21**, 171–173 (1998).

6. Dubois A, Sevely A, Boetto S, Delisle MB, Manelfe C. Clear-cell

meningioma of the cauda equine. Neuroradiology 1998;40:743-7..

7. Maxwell, M., Shih, S. D., Galanopoulos, T., Hedley-Whyte, E. T. & Cosgrove, G. R. Familial meningioma: Analysis of expression of neurofibromatosis 2 protein Merlin. *J. Neurosurg.* **88**, 562–569 (1998).

8. Cances C, Chiax Y, Karsenty C, Boetto S, Sevely A, Delisle MB,

Carriere JP. Clear cell meningioma: recurrent intraspinal tumor in a

child [in French]. Arch Pediatr 1998;5:758 - 62..

9. Heth JA, Kirby P, Menezes AH. Intraspinal familial clear cell meningioma in a mother and child. J Neurosurg (Spine 2) 2000; 93: 317–321

10. Jallo GI, Kothbauer KF, Silvera M, Epstein FJ. Intraspinal clear cell

meningioma: diagnosis and management: report of two cases. Neuro-

surgery 2001;48(1):218- 22..

11. Carrà S, Drigo P, Gardiman M, et al. Clear-cell meningioma in a 22-

month-old male: a case report and literature review. Pediatr Neurosurg

2001;34:264–7.

12. Florman J, Khoshyomn S, Tranmer B. Intraspinal clear cell meningioma:

diagnosis and management–report of two cases. Neurosurgery

2001;49:481. doi: 10.1097/00006123-200108000-00055.PMID: 11504136..

13. Yu KB, Lim MK, Kim HJ, et al. Clear-cell meningioma: CT and MR

imaging findings in two cases involving the spinal canal and

cerebellopontine angle. Korean J Radiol 2002;3:125–9..

14. Boet R, Ng HK, Kumta S, Chan LC, Chiu KW, Poon WS. Lumbosacral clear-cell meningioma treated with subtotal resection and radiotherapy. J Clin Neurosci. 2004;11(4):432-436. doi:10.1016/j.jocn.2003.10.014.

15. Chen MH, Chen SJ, Lin SM, et al. A lumbar clear cell meningioma with

foraminal extension in a renal transplant recipient. J Clin Neurosci

2004;11:665–7..

16. Payano M, Kondo Y, Kashima K, et al. Two cases of nondura-based clear cell meningioma of the cauda equina. APMIS 2004;112:141–7.

17. Oviedo A, Pang D, Zovickian J, et al. Clear cell meningioma: case report and review of the literature. Pediatr Dev Pathol 2005;8:386–90.

18. Epstein NE, Drexler S, Schneider J. Clear cell meningioma of the cauda

equina in an adult: case report and literature review. J Spinal Disord Tech 2005;18:539–43..

19. Dhall SS, Tumialán LM, Brat DJ, et al. Spinal intradural clear cell

meningioma following resection of a suprasellar clear cell meningioma.

Case report and recommendations for management. J Neurosurg

2005;103:559–63..

20. Liu PI, Liu GC, Tsai KB, Lin CL, Hsu JS. Intraspinal clear-cell meningioma: case report and review of literature. Surg Neurol. 2005;63(3):285-289. doi:10.1016/j.surneu.2004.03.013.

21. Jia Y, Zhong DR, Cui QC. Intraspinal clear cell meningioma: a case

report. Chin Med J (Engl) 2005;118:348–9..

22. Park SH, Hwang SK, Park YM. Intramedullary clear cell meningioma. Acta Neurochir (Wien). 2006;148(4):463-466. doi:10.1007/s00701-005-0695-z.

23. Vural M, Arslantaş A, Ciftçi E, Artan S, Atasoy MA. An unusual case of cervical clear-cell meningioma in pediatric age. Childs Nerv Syst. 2007;23(2):225-229. doi:10.1007/s00381-006-0181-9.

24. Salehpour F, Zeinali A, Vahedi P, Halimi M. A rare case of intramedullary cervical spinal cord meningioma and review of the literature. Spinal Cord. 2008;46(9):648-650. doi:10.1038/sj.sc.3102175.

25. Nakajima H, Uchida K, Kobayashi S, Takamura T, Yayama T, Baba H. Microsurgical excision of multiple clear cell meningiomas of the cauda equina: a case report. Minim Invasive Neurosurg. 2009;52(1):32-35. doi:10.1055/s-0028-1085455.

26. Colen CB, Rayes M, McClendon J Jr, Rabah R, Ham SD. Pediatric spinal clear cell meningioma. Case report. J Neurosurg Pediatr. 2009;3(1):57-60. doi:10.3171/2008.10.17668.

27. Tong-tong W, Li-juan B, Zhi L, Yang L, Bo-ning L, Quan H. Clear cell meningioma with anaplastic features: case report and review of literature. Pathol Res Pract. 2010;206(5):349-354. doi:10.1016/j.prp.2009.06.015.

28. Ko JK, Choi BK, Cho WH, Choi CH. Non-dura based intaspinal clear cell meningioma. J Korean Neurosurg Soc. 2011;49(1):71-74. doi:10.3340/jkns.2011.49.1.71.

29. Kobayashi Y, Nakamura M, Tsuji O, et al. Nondura-based clear cell meningioma of the cauda equina in an adult. J Orthop Sci. 2013;18(5):861-865. doi:10.1007/s00776-012-0217-9.

30. Zhang J, Shrestha R, Li J, Shu J. An intracranial and intraspinal clear cell meningioma. Clin Neurol Neurosurg. 2013;115(3):371-374. doi:10.1016/j.clineuro.2012.05.031.

31. Balogun JA, Halliday W, Bouffet E, Kulkarni AV. Spinal clear cell meningioma in a 3-year-old: a case report. Pediatr Neurosurg. 2013;49(5):311-315. doi:10.1159/000366452.

32. Wang XQ, Huang MZ, Zhang H, et al. Clear cell meningioma: clinical features, CT, and MR imaging findings in 23 patients. J Comput Assist Tomogr. 2014;38(2):200-208. doi:10.1097/RCT.0000000000000018.

33. Li P, Yang Z, Wang Z, et al. Clinical features of clear cell meningioma: a retrospective study of 36 cases among 10,529 patients in a single institution. *Acta Neurochir (Wien)*. 2016;158(1):67-76. doi:10.1007/s00701-015-2635-x

34. Kawasaki Y, Uchida S, Onishi K, Okanari K, Fujiki M. Pediatric nondura-based clear cell meningioma of the cauda equina: case report and review of literature. Br J Neurosurg. 2020;34(2):215-218. doi:10.1080/02688697.2018.1429565.

35. Inoue T, Shitara S, Ozeki M, Nozawa A, Fukao T, Fukushima T. Hereditary clear cell meningiomas in a single family: three-cases report. Acta Neurochir (Wien). 2018;160(12):2321-2325. doi:10.1007/s00701-018-3727-1.

36. Li J, Zhang S, Wang Q, et al. Spinal Clear Cell Meningioma: Clinical Study with Long-Term Follow-Up in 12 Patients. World Neurosurg. 2019;122:e415-e426. doi:10.1016/j.wneu.2018.10.064.

37. Zhang X, Zhang P, Wang JJ, et al. Intraspinal clear cell meningioma without dural attachment: A case report and literature review. Medicine (Baltimore). 2021;100(11):e25167. doi:10.1097/MD.0000000000025167.

38. Alsadiq MN, Albarbari ZS, Alshakhs F, Alduayji MA, Al-Umran S, Alenzi A. Spinal Clear Cell Meningioma: Atypical Clinical and Radiological Manifestations. Case Rep Surg. 2021;2021:9998399. Published 2021 May 27. doi:10.1155/2021/9998399.

39. Maamri K, Hadj Taieb MA, Trifa A, Elkahla G, Njima M, Darmoul M. Spinal clear cell meningioma without dural attachment: a case report and literature review. Radiol Case Rep. 2022;17(5):1760-1764. Published 2022 Mar 25. doi:10.1016/j.radcr.2022.02.052.
